# The effects of microglia-associated neuroinflammation on Alzheimer’s disease

**DOI:** 10.3389/fimmu.2023.1117172

**Published:** 2023-02-22

**Authors:** Cuicui Wang, Shuai Zong, Xiaolin Cui, Xueying Wang, Shuang Wu, Le Wang, Yingchao Liu, Zhiming Lu

**Affiliations:** ^1^ Department of Clinical Laboratory Medicine, Shandong Provincial Hospital Affiliated to Shandong First Medical University, Jinan, Shandong, China; ^2^ School of Medicine, Shandong University, Jinan, Shandong, China

**Keywords:** Alzheimer’s disease, neuroinflammation, microglia activation, therapy, β-amyloid

## Abstract

Alzheimer’s disease (AD) is defined as a severe chronic degenerative neurological disease in human. The pathogenic mechanism of AD has been convincingly elucidated by the “amyloid cascade hypothesis” with the main focus of the pathological accretion of β-amyloid (Aβ) peptides outside the cell. However, increasing evidence suggests that this hypothesis is weak in explaining the pathogenesis of AD. Neuroinflammation is crucial in the development of AD, which is proven by the elevated levels of inflammatory markers and the identification of AD risk genes relevant to the innate immune function. Here, we summarize the effects of microglia-mediated neuroinflammation on AD, focusing on the temporal and spatial changes in microglial phenotype, the interactions among microglia, Aβ, tau, and neurons, and the prospects and recent advances in neuroinflammation as a diagnostic and therapeutic target of AD.

## 1 Introduction

Alzheimer’s disease (AD) is the most common type of dementia, with its case number projected to be nearly doubled in Europe and tripled worldwide by 2050 (1). The brain atrophy, characterized by the accumulation of amyloid plaques and neuronal fibrillary tangles as well as the loss of neurons and synapses, are considered as the main pathological feature of AD. The “amyloid cascade hypothesis” is currently the most popular molecular mechanism regulating the AD pathogenesis, indicating that the misfolding and aggregation of β-amyloid (Aβ) result in a linear cascade of AD pathology. However, the addition of Aβ to the brains of healthy humans or mice does not cause the development of AD. Similarly, removal of Aβ from the brains of AD patients could not improve the medical conditions, suggesting that the “amyloid cascade hypothesis” alone can no longer fully explain the development of AD (2, 3). To date, it is well-known that neuroinflammation is responsible for the pathogenesis of AD. A total of more than 40 loci are confirmed as the target genes related to the late-onset AD (LOAD), with many related genes concentrated in glial cells. In particular, microglia have become a central topic in AD research (4). Microglia are natural immune cells in the central nervous system (CNS), participating in nerve development by phagocytosis and clearance of damaged neurons and synapses (5, 6). However, once activated to show the proinflammatory phenotype, microglia produce deleterious substances in patients with AD. This review focuses on neuroinflammation, microglial phenotype, and their interrelations with AD pathology, showing significant prospects of neuroinflammation and microglia as diagnostic and therapeutic strategies during the therapy of AD.

## 2 Neuroinflammation in Alzheimer’s disease

### 2.1 Alzheimer’s disease

AD is a neurodegenerative disease characterized by gradual deterioration of cognitive ability, with notably gradual death of neurons and often severe impairment of cognitive ability in memory and learning. It is well known that AD is a prolonged neurological disease with its development divided into three stages, i.e., preclinical stage, mild cognitive impairment (MCI) stage, and demented stage (7, 8). In about 20 years ago, the “amyloid cascade hypothesis” was proposed to explain the pathogenesis of AD based on the formation of abnormal Aβ plaques in different regions of the brain, suggesting that the abnormal accumulation of Aβ outside the cells was the main cause of the cascade effect leading to neuronal injury (9, 10). However, the accuracy of Aβ as a biomarker for AD is seriously challenged because Aβ is found in approximately 30% of cognitively normal elderly people (11). Similarly, the results of Aβ clearance as a treatment for AD patients are not satisfactory, indicating that the “amyloid cascade hypothesis” is unable to fully explain the molecular mechanism of AD pathogenesis (12, 13). Furthermore, another significant pathological change in AD is the intracellular aggregation of neuronal fibrillary tangles formed by tau aggregation (14). Moreover, several alternative hypotheses, i.e., the oxidative stress hypothesis, the cholinergic hypothesis, and the neuroinflammation hypothesis, are also proposed to provide significant insights into the pathogenesis of AD (15–17). Among these hypotheses, the neuroinflammation has been shown to be involved in the entire process of AD. Growing evidence suggests that persistent glial cell-mediated neuroinflammation is the primary cause for both neurodegenerative processes and cognitive deficits in AD patients. Inflammation is not only a passive outcome but also a contributor to the occurrence of AD, attracting increasing attention from medical scholars to further explore the important functions of neuroinflammation in the development of AD (18).

### 2.2 Neuroinflammation and its main risk factors

Neuroinflammation is a reaction of inflammation in the CNS due to pathological damage occurring in the peripheral or CNS, resulting in the production of proinflammatory cytokines, such as interleukin-1β (IL-1β), IL-6, IL-8, and tumor necrosis factor (TNF), chemokines, complement cytokines as well as some small molecule messengers, e.g., prostaglandins, nitric oxide (NO), and reactive oxygen species (ROS) (19). Generally, the acute inflammation in the brain exists as a defense against damage to the nervous system, infection, and other stimuli. However, with prolonged inflammation, the acute inflammation gradually becomes chronic to stimulate the nervous system. The chronic inflammation could continue to release various inflammatory cytokines, resulting in a proinflammatory response that is consistently stronger than the anti-inflammatory response, thus aggravating the inflammation, ultimately damaging the neurons and causing various pathological changes in the body (20). Endogenous bioactive lipids regulate a large number of cellular and molecular processes related to health and disease, especially during inflammation. All major endogenous bioactive lipids, i.e., eicosanoids, specialized pro-catabolic lipid mediators, lysophospholipids, and endocannabinoids, are closely associated with chronic inflammation (21). Although these molecules are originally described as neuromodulators/neurotransmitters, they have been characterized as important regulators of glial function (22) and will in turn disrupt tissue homeostasis to cause chronic damage, such as AD (23). Given the higher expression and production of inflammatory cytokines in microglia than those of other glial cells, microglia are considered the main sources of brain inflammation. Furthermore, the microglia have attracted increasing attention from the medical scholars due to their complex phenotypes. Moreover, microglia are also closely related to the loss of synapses, tau phosphorylation, and poor memory, making these biological processes increasingly the centers of the study of neuroinflammation in AD (24).

## 3 Microglia

Microglia are considered as the resident macrophages of the CNS, originated from mesoderm and migrating to the CNS during its development (25). On the one hand, microglia are known with an essential protective effect in the physiological processes of the CNS and are considered an important protection to maintain the homeostasis, constituting the frontline defense of the innate immune system and regulating the number of neurons in the CNS. During the body development, microglia promote neuronal survival and differentiation as well as circuit formation through brain-derived neurotrophic factor signaling; microglia also participate in synaptic formation related to learning. For example, studies have shown that CX3CR1 in microglia is essential for the survival of layer V neurons, while the microglia-derived IGF1 is a trophic factor for maintaining the neuronal survival, whereas the neurotrophic factors released by microglia could induce programmed cell death and maintain body stability (26). On the other hand, persistently activated microglia could contribute to the pathogenesis of neurodegenerative disease. For example, studies have shown that overactivated microglia could produce proinflammatory cytokines and chemokines at high levels, leading to neuronal dysfunction (27). Meanwhile, the abnormal activation of the complement-microglia axis is involved in the synaptic loss in the early stages of AD, which is closely related to the cognitive performance. Studies have shown that the inhibition of microglia complement receptors in a mouse model could reduce the degree of early synaptic loss (28, 29), while the microglia-mediated synaptic pruning may be the underlying mechanism of synaptic loss and memory impairment induced by long-term alcohol exposure (30).

### 3.1 Variations in phenotypic characters of microglia in AD

**Figure 1:**
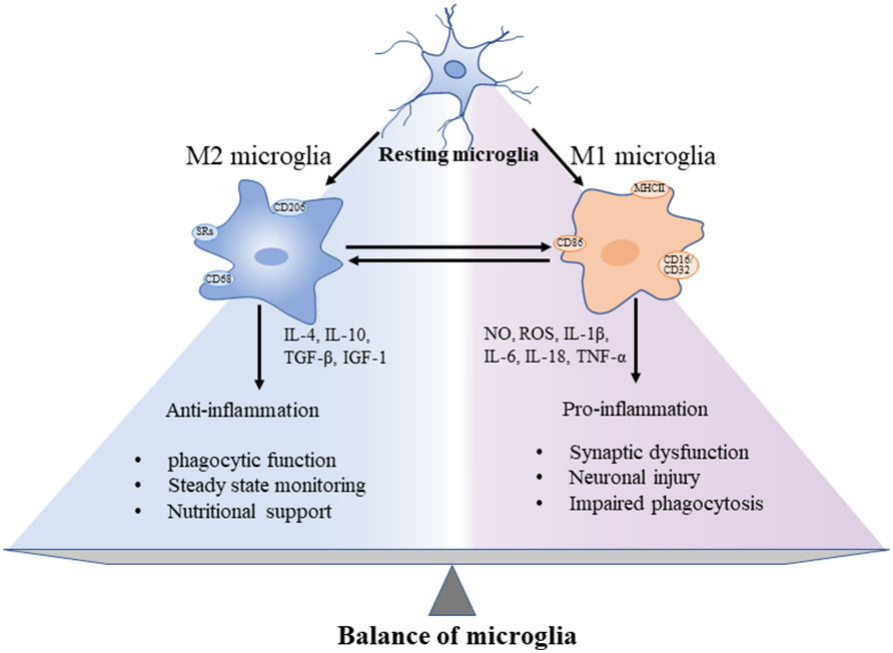
Different functions of microglia showing the “two-sided” characteristics of microglia and the balance between pro- and anti-inflammation in the progression of CNS neuroinflammation. These microglial phenotypes display different cell surface receptors and secreted factors, among which, by secreting anti-inflammatory cytokines and trophic factors to promote phagocytosis, maintain homeostasis, and nourish nerves. In addition, the surface tightening was activated to cause both synaptic dysfunction and neuronal damage.

In the rapidly changing environment of brain, precise differentiation of microglial phenotypes is essential to explore the pathological development of AD. The phenotypic characters of microglia could be divided into resting phase (M0) and activated phases (M1 and M2) (31, 32) (**Figure 1**). In the resting microglia, cytosol remains in a fixed position. The two-photon imaging of the cerebral cortex reveals that microglia at the resting phase are still functioning as a sensitive surveillance, with their cytosols and protrusions continuously monitoring changes in the microenvironment of the brain parenchyma (33). The cytosols of microglia at this phase are mostly highly branched, i.e., these cells are known as multipolar or branched microglia, which are particularly sensitive to the homeostasis disturbance of the internal brain environment. In AD, the brain homeostasis is impaired, accompanied by changes in both brain microenvironment and glial cell characteristics, while the microglia mainly show the activated anti-inflammatory phenotype M2, featured by the release of anti-inflammatory cytokines, including transforming growth factor-β (TGF-β), IL-4, IL-10, and IL-13, and enhanced phagocytosis (34). The persistent inflammation transforms microglia to the activated proinflammatory phenotype M1, which promotes inflammation and leads to increased concentrations of proinflammatory cytokines, including TNF-α, IL-4, IL-6, IL-12, and IL-18, accompanied by impaired phagocytosis (35), while the microglial cell body is enlarged, the processes become shorter, and the outline of microglial cells is changed to round (33). These polarized states differ in triggering stimuli, phenotypic markers, and expression of secretory mediators (**Figure 1**). In AD, the neuroinflammation is increased with the disease progression. Specifically, the neuroinflammation level reaches the first peak in the early development of AD, attributed to the initial anti-inflammatory response, and then a second peak during the transition from stage MCI to stage dementia, suggesting the change of microglia to a proinflammatory phenotype (36, 37). A longitudinal study revealed that microglial activation may attempt to repair damage during the initial stages of AD, while in LOAD, microglia could be harmful, producing proinflammatory molecules to cause neuronal damage (36). For example, microglia initially phagocytose apoptotic cells through Anexelekto (Axl) and Mer anti-inflammatory receptors (38), whereas the chronic microglial activation could lead to severe pathological changes and neurological complications. It is worth noting that although the M1/M2 nomenclature is rather useful in experimental paradigms, it has also been fiercely discussed, especially in microglia: in particular, M1- and M2-like phenotypes can be considered two extremes of a rather blurred distribution, rather than 2 distinct populations (39). Some authors strongly avoided this dichotomy, whereas others acknowledged that they still use it even with its limitations for lack of a better term (40). Dark microglial (DM) is a recently described phenotype, also known as “dystrophic” microglia, which was shown to precede the spread of Tau pathology (41, 42). Its dark appearance is detected under the high spatial resolution transmission electron microscopy (43). In the APP/PS1 mouse model, black microglia with complete and clear abnormal chromatin and dark appearance are observed. Compared with C57BL/6 mice, the density of black microglia in APP/PS1 mice is significantly increased and concentrated near both Aβ plaques and dystrophic neurites (44). The molecular mechanisms underlying the functions of different types of microglia remain to be explored.

#### 3.1.1 Variations of microglia in different stages of AD

Compared to microglia at steady state, hundreds of genes markedly up- and down-regulated in the early or late-response microglia have been revealed by the single-cell differential expression (SCDE) software packages. For example, a subpopulation of immune response-related genes, encoding the chemokines cytokine ligand 3 (CCL3) and cytokine ligand 4 (CCL4) as well as CXC chemokine ligand 16 (CXCL16) and macrophage migration inhibitory factor (MIF), are up-regulated in microglia subpopulations 1 week following p25 induction, while the expressions of genes encoding proteins such as Axl and apolipoprotein E (Apoe) were elevated in most late-responding cells except in 1-week CK-p25 cells (45). Previous studies revealed significant activation of microglia during MCI phase of rising load, followed by a decrease in microglia activation levels as Aβ load approached the AD levels, showing a positive correlation between increased inflammation levels and tau load when tau tangles are formed in Aβ-positive patients with mild cognitive impairment with prodromal AD (46). Moreover, microglia in brains of Braak stages V and VI undergo more noteworthy morphological changes compared to those from brains in earlier stages. The main manifestations include shortened or reduced branches of microglia, cytoplasmic fragmentation, and globular formation (47, 48).

#### 3.1.2 Variations of microglia in different spatial locations

Analysis of plaque-associated microglia and those not associated with plaques in the TgCRND mouse model of AD showed that the K1 current in microglia around plaques is significantly increased with evident morphological changes, while microglia not associated with plaques showed only slight changes (48, 49). Furthermore, microglia acquired in plaque-rich regions are found to show different transcriptional features than those of microglia derived from non-plaque regions (50, 51).

#### 3.1.3 Changes of microglia in response to different stimuli

Microglia have shown different transcriptional responses to Aβ peptide aggregates *in vitro* or *in vivo*, and even to exogenous oligomer Aβ (oAβ) or fibrillar Aβ (fAβ) (52), with the expressions of RNAs related to cell cycle and phagocytosis increased by oAβ and fAβ, respectively, and they responded differently depending on the timing of the stimulus. Furthermore, research has shown that in mouse models of AD, the microglial proliferation is increased linearly with the development of the disease, whereas the Aβ load reached a plateau in the early clinical stages of the disease (53, 54), suggesting that microgliosis at this stage is associated with the tau tangles.

#### 3.1.4 Changes of microglia in different pathological models

Although the results of postmortem of microglia in the brains of cognitively normal participants have shown similar levels of gene expression profiles to those of mice, the genes that regulate microglia during aging are only partially overlapping between humans and mice (55). This variation could partially explain the frequently failed translation of anti-inflammatory strategies into clinical practice. As a microglial surface receptor, TREM2 has been found to have inconsistent effects *in vitro* and *in vivo*, with its expression regulated by inflammation (56). Studies have shown that the TREM2 expression is decreased during acute inflammatory responses *in vitro* (57) and is increased in both AD patients (58) and mouse models of Aβ and Tau pathology (59). Therefore, it is necessary to distinguish between acute and chronic microglial activations in order to elucidate the molecular mechanisms underlying the TREM2 expression. It is essential to detect microglia and respond to neural deformation signals. The *in vivo* studies have shown that TREM2 is widely involved in the uptake of Aβ by microglia, whereas the *in vitro* studies have shown inconsistent results (56, 57).

### 3.2 Activation stimulation of microglia

Studies have shown that the microglial phenotype regulation depends primarily on the relationship between pattern recognition receptors (PRRs) and molecules released from the surrounding cells (60). In AD, these PRRs could sense Aβ, pathogen-associated molecular patterns (PAMPs), or other damage-associated molecular patterns (DAMPs) to influence the microglial phenotype. Microglia activation is characterized by enhanced responsiveness to various inflammatory factors or injury stimuli. After the microglia perceive the DAMPs and PAMPs through toll-like receptors (TLRs), retinoic acid-inducible gene-I (RIG-I)-like receptors (RLRs), nucleotide-binding oligomerization domain (NOD)-like receptors (NLRs), or other PRRs, the signaling through PRRs could trigger an inflammatory response and proinflammatory cytokine secretion (**Table 1**). Generally, these activated microglia are called disease-associated microglia (DAM) (73–77). Furthermore, the Aβ binding particularly to TLR4 has been shown to activate microglia (63, 78). The main receptors expressed by activated microglia include scavenger receptors (SRs), TLRs, triggering receptors in myeloid cells 2 (TREM-2), CD14, CD47, Fcγ receptors (FcγRs), CD200 receptor (CD200R) and receptor for advanced glycation end products (RAGE). Specifically, CD14, TLR2, and TLR4 are required for fAβ-stimulated microglial activation (79), though the molecular mechanisms regulating this microglial activation remain unclear. However, this sustained activation of microglia is adverse because the extended activation of TLR2 and TLR4 in microglia could induce the secretion of Aβ (80). TREM2 signaling appears to be required for the maintenance of microglial metabolism, while the deficiency of TREM2 leads to impaired microglial survival, migration and, phagocytosis (81). In addition, the mild activation of TREM2 has shown anti-inflammatory function to inhibit TLR4 receptor-induced proinflammatory response (82–84). However, the role of TREM2 in the inflammatory process is still controversial. For example, the injection of AD-Tau into the brain of mice with amyloidosis to induce Aβ-dependent Tau deposition showed that TREM2 consistently activated microglia and aggravated Tau pathology as well as neurodystrophy (85). Furthermore, other Aβ peptides, fibrils, and APPS are also potent activators of microglia (86). In AD pathology, the PRRs bind to different types of Aβ with different affinities to initiate the activation of glial cells, causing accumulation of more microglia and then increasing the density of microglia (87). Moreover, Aβ could activate the NF-KB-dependent pathways to bind to the surface of microglia to activate extracellular signal-regulated kinase (ERK) and mitogen-activated protein kinase (MAPK) pathways, ultimately triggering the proinflammatory gene expression (88, 89). An autopsy study of the temporal neocortex in 15 control subjects without dementia and 91 AD patients has indicated that in the postmortem brain of AD patients, even after the Aβ plaque growth ends, the density of microglia is increased linearly and positively correlated with NFTs burden, which is consistently observed in the non-amnesic AD variant of primary progressive aphasia (54, 90). Inconsistent with other studies, the activation of Wnt-3a in primary microglia could promote microglia into the proinflammatory state of AD (91–93). Furthermore, the exogenous interfering factors, e.g., lipopolysaccharides (LPS), injected intraperitoneally in 5×FAD mice and APP23 mice are subsequently observed in the microglial hyperresponsiveness around the dense core plaque (94). Due to its significant biological importance, the activation of microglia caused by inflammation which is induced by overactivated microglia has attracted increasing attention to explore the molecular mechanisms regulating the microglial phenotypic variations.

**Table 1:** Microglia receptors associated with Alzheimer’s disease (AD).

Sensor	Model system	Role in AD pathogenesis	Reference
TLRs			
TLR2	APP/PS1 mice	Inhibition of TLR2 activation increases the Aβ deposition; impaired recognition; increased neuroinflammation	(61, 62)
	AD-TLR2KO	TLR2 genomic loss evokes exacerbation of white matter injury and worsens neurobehavioral function	
TLR4	TLR4M Tg mice	Increase of Aβ deposition and soluble Aβ42 in the brain of TLR4M Tg mice and decrease of IL-1b, CCL3, and CCL4 expressions in cognitive function and hippocampus compared to TLR4W Tg mice; TLR4 mutations reduce the Ab-induced IL-1b, CCL3, and CCL4 expressions in monocytes	(63, 64)
	TLR4w AD mice and TLR4m AD mice	Activation of microglia and up-regulation of TLR4-dependent cytokines	
Inflammasome sensors			
NLRP1	Human neurons	NLRP1 inflammasomes expressed in neurons of human central nervous system are involved in axonal degeneration and cognitive dysfunction by activating Casp1 and Casp6	(65)
NLRP3	NALP3-deficient mice	Involved in lysosomal damage and release of tissue proteinase B; activation of inflammation and tissue damage in AD	(66, 67)
	APP/PS1 mice	Promotion of microglia conversion to M2 phenotype and reduction of Aβ deposition	
Other cell surface receptors			
TREM2	TREM2-WT) or TREM2-R47H	Reduction of Aβ seeding and inhibition of disease-associated microglia	(68, 69)
	TREM2 KO and WT C57BL/6J	TREM2 deficiency promotes tau transport, distribution, and seeding through microglial cell exosomes	
CD200	Primary astrocytes and microglia	Prevents Meth-induced microglial activation and glutamate toxicity reaction	(70, 71)
	CD200 +/+ and CD200 −/− mice	MPTP experimental mouse model	
CD32	C57BL/6 mice	Induce proinflammatory signaling	(72)

## 4 Interactions among microglia, Aβ, tau, and neurons

**Figure 2:**
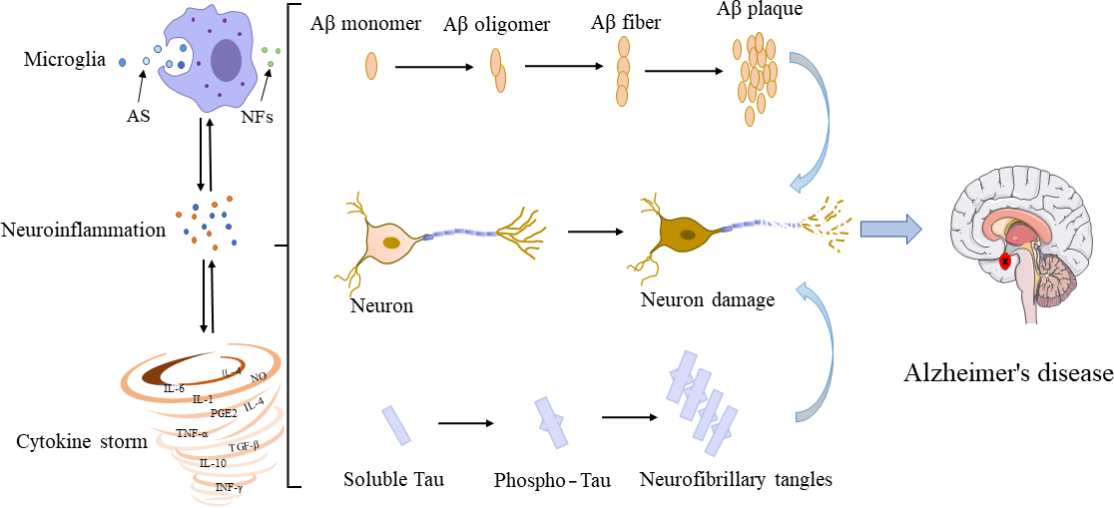
Interconnections among microglia, Aβ, tau, and neurons. In their early developmental stages, microglia enhance the phagocytic capacity and generate phagocytoma abnormal material, such as cell debris and misfolded proteins. Subsequently, M2 microglial cells are promoted to release proinflammatory mediators, leading to an inflammatory storm. In this process, the amyloid monomers gradually accumulated to form amyloid plaques. Meanwhile, free tau proteins are phosphorylated and aggregated in neurons to form neurofibrillary tangles. The accumulation of Aβ plaques and the formation of neurofibrillary tangles could positively feedback to microglia to promote the activation of microglia, leading to impaired ability to phagosome foreign objects. AS, abnormal substances (i.e., cell debris and misfolded protein); NFs, neurotrophic factors.

In the 1990s, microglia were proved to have evident interactions with Aβ and tau (**Figure 2**). Although their interconnections have been extensively investigated, the molecular mechanisms underlying their interconnections remain unclear. The glial cells and neurons interact in a synchronous manner to facilitate the occurrence and development of AD. The microglia influence the neuronal activity by direct physical contact or by releasing paracrine signal (94). Studies have shown that the overproduction of Aβ by neurons could stimulate the NF-κB pathway in astrocytes to trigger an increase in the extracellular expression of complement C3 and then to reverse to an adverse effect on both neurons and microglia, leading to neuronal damage and microglia activation (95, 96). Inconsistently, the activated microglia induce the A1 neurotoxic astrocytes to cause the loss of the ability of A1 astrocytes to foster neuronal survival, growth, synaptic initiation and phagocytosis, and induce the neuronal and oligodendrocyte death (97). Notably, microglia are recruited to neurons 7 days before their elimination with the neuronal loss reduced (98). In transgenic mice, microglia rapidly respond to plaque formation through the process of expansion, and then migrate to plaques and accumulate around amyloid plaques. The size of plaques varies with microglia volume, i.e., larger plaques are generally surrounded by larger microglia (99). In young APPswe/PS1d9xYFP transgenic mice, a dynamic plaque-forming process is detected in 24 hours and microglia are activated and recruited to the area in 1-2 days after the appearance of the new plaque, which is followed by progressive neuropathy (100). Among all types of Aβ peptides, the microglia induced by small oligomers of Aβ have shown stronger neurotoxicity relative to the relatively large oligomers, ultimately causing more severe neuronal death (101). Furthermore, the *in vivo* imaging has revealed that microglia could function as transporters to help Aβ propagate in unaffected tissues (102). Moreover, the chronically activated microglia around Aβ plaques could release inflammatory factors while engulfing plaques, leading to excessive activation of microglia and ultimately aggravating the disease (103).

The relationships between Aβ and neuroinflammation have been extensively studied to provide support of the “amyloid cascade hypothesis,” while studies on the relationships between microglia and tau are sparse. Typically, tau mediates microtubule stability and aggregation by interacting with tubulin; in AD, tau is transformed into an insoluble form once it is dissociated from microtubules, further resulting in the formation of neurofibrillary tangles to increase the levels of reactive microglia surrounding tau (104, 105). The snRNA-seq analysis of nuclei has revealed different expression profiles in the microglia population, with AD2-microglia showing a strong correlation between the abundance and phosphorylated tau burden in the tissue, which are more enriched in tau pathological samples (106). Studies have shown that neurodegenerative microglia (MGnD) overproduce p-tau^+^ EVs in parallel with both reduction of Aβ plaques and removal of NP tau, which is suggested as a possible mechanistic link for the association between increased Aβ deposition and tau propagation in APP mutant knock-in homozygote (AppNL-G-F) mice (107). However, recent research has indicated that immunosuppressors attenuate tau pathology by inhibiting microglial activation in a mouse model of tau pathology (P301S), providing experimental evidence to link early microglial activation with tau pathology (108). To date, the pathogenic mechanism underlying the microglial promotion of tau deposition remains unclear. It is proved that tau could activate the NF-KB signaling pathway, driving microglia-mediated seeding and spreading of tau, as verified by single-cell RNA-seq analysis (109). Furthermore, studies have shown that the polyglutamine binding protein 1 (PQBP1) in microglia first senses the exogenous tau 3R/4R proteins through direct interaction and then provokes innate immune reactions through triggering the cyclic GMP-AMP synthase (cGAS) in the stimulator of interferon gene (STING) pathway, while PQBP1 is required for sensing the tau-induced NF-KB translocation, NF-κB-dependent transcription of inflammatory genes, brain inflammation, and ultimately cognitive impairment in mice (110). Given the complex interconnections among glial cells, Aβ, tau, and neurons, the communications among these units generate the positive feedback in the inflammatory environment of AD, causing a disordered and self-amplified inflammatory response.

## 5 Treatment strategy of AD

To date, the therapeutic interventions to lower the Aβ level have yielded unsatisfactory results, and even the oral administration of β-site amyloid precursor protein–cleaving enzyme 1 (BACE-1) inhibitors that block Aβ production fails to reduce the cognitive or functional decline in the patients suffering mild to moderate AD (12). Likewise, many clinical trials have not been successful in eliminating various forms of Aβ and tau from the brains of AD patients (13), suggesting that the plaque removal may not be an ideal strategy for treating symptomatic AD, although it could still be a feasible preventive treatment. In contrast, targeting the microglia for therapeutic intervention against neuroinflammation exacerbated by overactivated microglia represents a potentially promising therapeutic strategy for AD. Therefore, it is important to identify the actions of microglia in the process of AD development in order to intervene and treat AD by the following methods: first, suppressing microglial priming in the pre-disease phase; second, limiting the proinflammatory response of microglia; and third, modulating and transforming the phenotypes of microglia to an anti-inflammatory phenotype.

### 5.1 Suppression of the microglial priming

Various factors induce imbalances of the CNS, e.g., aging, systemic inflammation, or stress, leading to the initiation of microglia and causing microglia to show strong susceptibility and responsiveness toward inflammatory stimuli. Therefore, a potential treatment strategy for AD could be to inhibit pre-disease microglial priming. Studies have shown that the middle-aged AD patients with obesity and insulin-resistance are prone to inducing inflammation, while statins exhibit a protective effect against this injury, preventing the initiation of microglia (111, 112). Furthermore, supplementation with folic acid and omega-3 fatty acid has also been shown to intervene the neuroinflammation and reduce the level of inflammation in both the cerebrospinal fluid and blood of individuals with AD (113, 114).

### 5.2 Inhibition of proinflammatory response

The factor of NF-κB has been implicated in physiological processes related to signaling, cognition, and memory in CNS. This factor could be activated by oxidative stress, neuroinflammation, and other factors, leading to CNS dysfunction in AD patients (115). The production of ROS initiates the enzymatic activities of IKKb to phosphorylate the heterodimer of NF-κB, which is an inhibitor of kappaB (IκB), leading to its degradation *via* the ubiquitin-proteasome pathway, while the dissociation of IκB from the dimer could initiate the NF-κB influx into the nucleus (116). It is indicated that the activation of NF-κB stimulates the BACE1 expression and Aβ processing, which is probably a novel molecular mechanism regulating the AD progression (117). It has been discovered that inhibition of NF-κB activation by antioxidants, polyphenols, and non-steroidal anti-inflammatory drugs (NSAIDs) is essential to reduce the load of Aβ (118). For example, Tanshinone I is used to inhibit the synthesis and expression of several proinflammatory M1 mediators, significantly suppressing the LPS-induced activation of NF-κB in microglia (119), while Forsythia B regulates the neuroinflammation in APP/PS1 mice, effectively ameliorating cognitive function, reducing the accumulation of Aβ and tau, and attenuating glial cell activation in the hippocampus (120). Similarly, when the NF-kB signaling pathway is inhibited, the release of pathogenic tau by primary microglia could be reduced to rescue the autophagy defect of microglia and reduce the neurotoxic reaction (109).

The NOD-like receptor thermal protein domain associated protein 3 (NLRP3) are highly enriched in microglia comparison to both NLRP1 and NLRP2 (121). Therefore, it is worth further exploring the potential application of NLRP3 in the diagnosis and treatment of AD compared with other types of inflammatory vesicles. Indeed, there is ample evidence to show that the Aβ level in brains of APP/PS1 or TgCRND8 mice could be reduced with the memories modified following the use of small molecular inhibitors of NLRP3 inflammatory vesicles or knockdown (KO) of NLRP3 expression (122, 123). Furthermore, inhibition of NLRP3 inflammasome reduces IL-1β secretion and limits the injury to neighboring tissues, while IL-1β is thought to enhance the expression of s100β protein, which is closely associated with AD (124, 125). For example, benzylisothiocyanate (126), stavudine (127), and artemisinin (128) all have shown therapeutic effects on AD mice by inhibiting NLRP3 activation.

### 5.3 Regulation of phenotypic variations in microglia

Microglia support the neuronal function by removing toxic damage during the early stages of AD. Interference with the activation of microglia could allow their anti-inflammatory effect to persist for a long time. For example, the activated PPPAR-γ could inhibit the inflammatory response, while in mouse model of AD, treatment with PPAR-γ activators, e.g., pioglitazone and rosiglitazone, could switch microglia to show anti-inflammatory phenotype with phagocytosis (37, 129–131), which is probably related to the phagocytosis of amyloid deposits.

## 6 Conclusions and future prospects

The microglia response to injury suggests the significant potential of microglia used as a diagnostic marker for the development and progression of AD. The release of proinflammatory factors could be detected in the olfactory bulb (OB) of 2-month-old 5×FAD mice, showing significant microglial activation and morphological variations (132), suggesting that microglia-mediated OB neuroinflammation could serve as an early biomarker of AD. Even before the positive β-amyloid pathology is shown, the increased microglial activation is associated with defective NREM rapid-sleep spindle expression in the frontal cortex (24), providing evidence for the earlier diagnosis of AD. The restraint of microglia activation and the associated proinflammatory mediators are diagnostic and therapeutic targets of AD. Because microglia have different phenotypes with varied functions, it will be important to further explore the cause of the microglia activation detected in the initial phase of AD, either the anti-inflammatory M2 phenotype attempting to clear Aβ and protect neurons or an M1 phenotype leading to the neuronal damage. The interventions targeting the proinflammatory phenotype of microglia in the earlier phase of AD could be a potentially efficient strategy to treat AD.

## Author contributions

ZL, CW, and XC jointly completed the conception and writing of the review. SZ, XW, and SW completed the picture drawing of the review. YL and LW provided assistance for literature search and manuscript editing. All authors contributed to the article and approved the submitted version.
